# New Insights into Nuclear Magnetic Resonance (NMR) Spectroscopy

**DOI:** 10.3390/molecules30071500

**Published:** 2025-03-27

**Authors:** Ioannis P. Gerothanassis, Teobald Kupka

**Affiliations:** 1 Department of Chemistry, University of Ioannina, GR-45110 Ioannina, Greece; 2 Institute of Chemistry, University of Opole, 48, Oleska Street, 45-052 Opole, Poland

NMR spectroscopy is playing an increasing role in chemistry, biology, material sciences and related disciplines. The main purpose of this Special Issue, which includes nine (9) original articles and two (2) review articles, is to explore newer aspects of NMR spectroscopy. The articles can be classified into six categories: (i) experimental methods [1,2]; (ii) machine learning approaches [3,4]; (iii) quantum chemical calculations of NMR parameters [5,6]; (iv) applications in organic chemistry [7]; (v) investigation of protein–ligand interactions [8]; and (vi) applications in metabolomics [9,10,11].

In NMR spectroscopy, referencing is of great importance for accurately determining chemical shifts, and this has been widely discussed in the literature [12,13,14,15,16]. The most common methods of referencing in proton and ^13^C NMR spectra are based on the use of TMS as an internal standard or the solvent’s residual peak as a secondary reference. In this Special Issue, Nazarski [1] critically reviewed the use of deuterated organic solvents without TMS to report ^1^H and ^13^C NMR data. An overview of chemical shift data in the literature revealed discrepancies of up to 1.9 ppm for ^13^C in CDCl_3_. It was shown that the δ scale strongly depends on the concentration and structural characteristics of the analyte and solvent selected. The use of a single solvent and dilute solution is recommended. Recently, Nazarski extended his study and analyzed the use of DSS in D_2_O as a secondary reference for ^1^H and ^13^C NMR [17].

The assignment of ^13^C NMR signals to individual atoms of a molecule under investigation has been an important step in organic chemistry laboratory work [18,19,20]. In this Special Issue, Bigler et al. [2] reported a broadband proton test sequence that can be utilized to record multiplicity-edited and quaternary carbon spectra. The pulse sequence results in high tolerance over a wide range of ^1^J_CH_ and very good suppression of residual CH_n_ signals in the quaternary carbon, Cq, spectrum. Although the proposed method suffers from sensitivity relative to standard heteronuclear inverse 2D ^1^H-^13^C HSQC and HMBC experiments [13], its excellent resolution and short experimental time could prove advantageous in the analysis of mixtures in metabolomics [9].

The rapid development of the NMR technique was based on the introduction of radiofrequency pulses and the subsequent Fourier transform of the voltage signal from the time domain to the frequency domain [20,21]. In this Special Issue, Perez Varela et al. [3] presented a practical approach involving a signal processing formalism for the proper alignment of NMR signals, with an audio output, for various 1D NMR/FID spectra. The visual harmony of FID signals, formed by a time-domain NMR response, was revised and modernized as a tool capable of recognizing aromatics and olefinic or aliphatic protons. This auditory feature was integrated into the Mnova program, a widely used NMR platform.

There is a direct correlation between the geometrical structure of a chemical compound and its proton and carbon NMR spectra. However, the complexity of many experimental spectra, including biomolecules, could be unraveled with the support of theoretical techniques. The prediction of ^13^C chemical shifts using database algorithms has been widespread in recent decades [22,23]. In this Special Issue, Duprat et al. [4] reported a graph machine tool to accurately estimate ^13^C chemical shifts of benzene-type compounds. A database of 10,577 chemical shifts from 2025 structures containing up to 10 non-carbon atoms (H, O, N, S, P, Si and halogens) was used. The predictive capability of the graph machine model, with a root-mean-square error (RMSE) of 0.9 ppm, was shown to be superior relative to open-source methods in the literature, with an RMSE of 3.4 to 1.9 ppm.

Quantum-chemical NMR methods have been extensively utilized in structural elucidation, conformational analysis, the modeling of noncovalent interactions and the quantification of the agreement between experimental data and theoretical calculations [24,25,26,27]. In this Special Issue, Hansen [5] summarized recent developments in the use of DFT calculations of NMR parameters in solution and in solid state. Emphasis was given to the accurate determination of OH and NH protons in hydrogen-bonded systems and the reassignment of experimental chemical shifts of complex natural products, including correct stereochemical assignment in the presence of several stereo-centers. Tautomerism also has widespread applications, in which structures of non-isolable tautomers can be calculated with the synergy of NMR chemical shifts and coupling constants through DFT calculations.

In this Special Issue, Venianakis et al. [6] investigated structural models of monounsaturated and ω-3 polyunsaturated free fatty acids (FFAs) in solution (CDCl_3_ and DMSO-d_6_) and in liquid state. The variable temperature and concentration, δ(^1^H), of the COOH group, transient 1D NOE experiments, and the DFT calculation of δ(^1^H) in CDCl_3_ were interpreted in terms of low-molecular-weight aggregates of classical dimeric intermolecular hydrogen bonds of the carboxylic groups in antiparallel structures. In DMSO-d_6_, the carboxylic groups form strong intermolecular hydrogen bonds with a single molecule of DMSO. DFT calculations of strongly deshielded labile protons, such as carboxylic acid -COOH, phenol OH, alcohol OH, amine NH and peptide CONH groups, can provide a general method for solvent-dependent structural and conformational analyses [28].

[3+2] cycloadditions are a cornerstone in the synthetic organic chemistry of complex molecular systems [29]. In this Special Issue, Malucka and Vilkova [7] investigated [3+2] cycloadditions in acridine-based dipolarophiles with unstable nitrile N-oxides. The complete structural characterization of the isolated regio-isomeric compounds was achieved with a variety of 1D and 2D (COSY, NOESY, HSQC-TOCSY and HMBC) NMR techniques. The regio-selectivity of the reactions was found to be mainly controlled by the electronic factors of the alkene on the basis of their ^13^C chemical shifts.

The saturation transfer difference (STD) and its variants [30,31], alongside transfer NOEs for pharmacophore mapping (INPHARMA) NMR [32,33] in conjunction with computational methods, have been extensively used to investigate ligand binding modes, even in proteins with multiple binding sites [34]. Furthermore, 2D NOE experiments, although much less sensitive than the STD, can also provide intra-NOE connectivities, which are a valuable tool for investigating conformational changes in ligands upon binding to macromolecules [35]. In this Special Issue, Alexandri et al. [8] performed 2D INPHARMA competition experiments of free fatty acids (FFAs) with drugs (ibuprofen and warfarin) of known X-ray structure determination. Using inter-ligand NOEs and docking calculations, they identified two conformational states of the polyunsaturated free fatty acids, DHA/EPA, due to the presence of two anchoring polar groups of amino acids within the FA7 binding pocket. The exceptional flexibility of EPA and DHA and the interconversion among the two binding sites are the main reasons that the location and conformational states of FFAs at the binding site of warfarin (FA7) could not be determined accurately despite numerous X-ray structural studies [36,37,38].

Extra virgin olive oil (EVOO) is a high-quality product that is prone to adulteration. Therefore, its authenticity and traceability have been extensively investigated with numerous analytical techniques, which usually involve separation steps and sensitive detectors. NMR spectroscopy has the advantage of being able to detect and quantify a variety of organic compounds simultaneously [39,40,41]. In this Special Issue, Rotondo et al. [9] reported a new NMR method using ^1^H double-pulsed gradient spin-echo and ^1^H decoupled ^13^C NMR with data processing to quantify the free fatty acid acidity, fatty acid ester composition and total phenolic content of EVOOs. Data collection and statistical processing are discussed in relation to climate change and pedoclimatic conditions.

Human metabolomics, which are designed to link small-molecular-weight metabolites (less than ~1500 Da), biochemistry/molecular biology data and clinical data, have been extensively utilized over the last few decades using primarily NMR and MS spectral data [42]. The discovery of novel disease biomarkers and the translation of metabolomic signatures into clinical practice are significant challenges in the field [43]. Short structure (SS) is one of the most common disorders in pediatrics [44] and can be attributed to endocrine causes, genetic syndromes, systemic diseases and idiopathic stature (ISS). In this Special Issue, Chang et al. [10] performed a comprehensive NMR-based metabolomic analysis to identify biomarkers of different types of SS. The authors were able to identify metabolite differences between growth hormone deficiency (GHD) and idiopathic short stature (ISS) in preadolescents and adolescents, which could be important in individualized clinical prognosis and treatment.

The dysregulation of lipid metabolism plays a significant role in the pathogenesis of coronary heart disease (CHD), which is among the leading causes of mortality worldwide [45]. In this Special Issue, Kastani et al. [11] used NMR-based lipidomics to investigate differences in the red blood cell (RBC) membrane lipidome between patients with CHD and those with normal coronary arteries. Targeted and non-targeted lipidomics analyses revealed significant alterations in the RBC membrane for CHD patients and thus can be used to investigate the progression of atherosclerosis, even at proatherogenic stages.
